# Cultural Perspectives on the Efficacy and Adoption of the Crohn’s Disease Exclusion Diet across Diverse Ethnicities: A Case-Based Overview

**DOI:** 10.3390/nu16183184

**Published:** 2024-09-20

**Authors:** Rotem Sigall Boneh, Sowon Park, Maria Soledad Arcucci, Marta Herrador-López, Chen Sarbagili-Shabat, Nitzan Kolonimos, Nicolette Wierdsma, Min Chen, Einat Hershkovitz, Eytan Wine, Johan Van Limbergen

**Affiliations:** 1 Pediatric Gastroenterology and Nutrition Unit, The E. Wolfson Medical Center, Holon 8820027, Israel; ibd.chen@gmail.com; 2 Tytgat Institute for Liver and Intestinal Research, Amsterdam Gastroenterology Endocrinology and Metabolism, University of Amsterdam, 1105 AZ Amsterdam, The Netherlands; j.e.vanlimbergen@amsterdamumc.nl; 3 Amsterdam Public Health Research Institute, University of Amsterdam, 1105 AZ Amsterdam, The Netherlands; 4 Severance Pediatric IBD Research Group, Department of Pediatric Gastroenterology, Hepatology and Nutrition, Yonsei University College of Medicine, Severance Children’s Hospital, Seoul 03722, Republic of Korea; sowon81@yuhs.ac; 5 Gastroenterology Service, Department of Pediatrics, Hospital de Niños Ricardo Gutierrez, Buenos Aires C1425EFD, Argentina; maria.arcucci@hospitalitaliano.org.ar; 6 Pediatric Gastroenterology, Hepatology and Liver-Intestinal Transplantation Unit, Department of Pediatrics, Hospital Italiano de Buenos Aires, Buenos Aires C1199ABB, Argentina; 7 Pediatric Gastroenterology and Nutrition Unit, Hospital Regional Universitario de Málaga, 29011 Málaga, Spain; herradorlopezm@gmail.com; 8 The Faculty of Medicine, Tel-Aviv University, Tel-Aviv 6997801, Israel; 9 IBD Unit, Gastroenterology Institute, Haemek Medical Center, Afula 1834111, Israel; nitzanver@gmail.com (N.K.); einat.h1@gmail.com (E.H.); 10 Department of Nutrition and Dietetics, Amsterdam University Medical Centers, 1105 AZ Amsterdam, The Netherlands; n.wierdsma@amsterdamumc.nl; 11 Amsterdam Gastroenterology Endocrinology Metabolism, 1105 AZ Amsterdam, The Netherlands; 12 Division of Pediatric Gastroenterology, Department of Pediatrics, University of Alberta, 11405 87th Ave., Edmonton, AB T6G 1C9, Canada; min.chen@albertahealthservices.ca (M.C.); wine@ualberta.ca (E.W.); 13 Department of Paediatric Gastroenterology and Nutrition, Amsterdam University Medical Centers, Emma Children’s Hospital, 1105 AZ Amsterdam, The Netherlands

**Keywords:** Crohn’s Disease (CD), Crohn’s Disease Exclusion Diet (CDED), dietary therapy, culture, ethnicity, dietitians

## Abstract

Background: The Crohn’s Disease Exclusion Diet (CDED) is a whole-foods regimen that has demonstrated efficacy in inducing remission among children and adults with mild-to-moderate disease. While initial studies predominantly originated from Israel, recent years have witnessed the expansion of experiences to diverse cultures, culminating in the recognition of CDED in the latest ESPEN guidelines. However, implementing dietary therapy poses significant challenges across various cultures, necessitating adaptations. Aim and Methods: This case-based study aims to present the collective experience from different cultures, shedding light on the encountered challenges and the corresponding solutions devised to surmount them by convening healthcare providers (dietitians and physicians across six countries and eight cultural settings) with extensive experience in utilizing the CDED. Results and Conclusions: Our findings underscore the efficacy of CDED across diverse cultural contexts and emphasize the pivotal role of dietitians in tailoring the diet to accommodate patients’ cultural behaviors and traditions. We highlight challenges encountered and delineate strategies for overcoming them by customizing the diet and offering tailored guidance. Additionally, we provide insights into implementing CDED in various regions through adjusted recipes and personalized counseling from dietitians. This study contributes to the growing body of literature on CDED, and offers practical guidance for its effective adoption in diverse cultural settings.

## 1. Introduction

In recent years, the role of diet in the management of inflammatory bowel disease (IBD) has increased dramatically, with the recognition of Exclusive Enteral Nutrition (EEN) as a first-line therapy according to current guidelines [1,2]. The Crohn’s Disease Exclusion Diet (CDED) has emerged as a whole-foods approach aimed at reducing inflammation by minimizing exposure to pro-inflammatory ingredients that may affect the intestinal barrier or microbiome [3]. CDED was suggested as a better tolerated approach to EEN to reduce inflammation, and was first described in 2014 among Israeli children and adolescent cohorts [4]. CDED was designed to be adaptable across various countries and cultures, incorporating simple and internationally accessible foods, but was mostly focused on “Western” populations. Fruits and vegetables were particularly selected for their fiber content, palatability, and availability. The standardization of the diet recommended certain “recommended foods” to ensure a balanced intake of essential nutrients supporting growth and microbiome health, although their necessity for dietary success remains uncertain [5,6].

The pivotal randomized controlled trial (RCT), which included Israeli and Canadian cohorts, facilitated the adaptation of CDED by other countries [5]. Table 1 outlines the experiences of CDED implementation across different nations in the literature to date.

To date, the available research published on experiences with the CDED has mostly been conducted in various countries, including Israel, Canada, the United States, and Argentina, as well as diverse European countries, such as Spain, Ireland, Sweden, Croatia, and Poland, with ongoing studies in France and The Netherlands [5,7,8,9,10,11,12,13,14,15,16,18]. Notably, the expansion of CDED into diverse cultural contexts has allowed participation from varied populations and ethnicities, with ethnicity never serving as an exclusion criterion.

Dames et al. underscored the importance of diversifying recruitment in clinical dietary studies, and proposed tailoring dietary interventions to specific communities [21]. They outlined challenges and potential solutions to enhance inclusivity.

While CDED is gaining momentum, with widespread use worldwide, challenges remain regarding the implementation of this dietary intervention. In contrast to pharmacological therapies, diet-based therapies require adaptations for various cultures, economic conditions, and availability. Building on the accumulating experience in adapting CDED to different cultures, this study convened dietitians and physicians from various countries, cultures, and ethnic backgrounds to share their insights on CDED, including clinical aspects, challenges encountered, and strategies employed based on their diverse experiences with specific cases and overall strategies. Emphasizing experiential insights, our goal is to inform future research and clinical practices in managing CD globally, rather than drawing broad scientific conclusions. Table 2 presents the experiences of each participating center in treating patients with CDED.

Hence, the objective of the present study was to collect insights from various experienced healthcare providers from diverse regions, cultures, and ethnicities, sharing their clinical experience and the adjustments necessary for successful treatment, as well as strategies for overcoming obstacles in the pursuit of the better implementation of dietary therapy for CD.

## 2. Materials and Methods

Healthcare providers (HCPs) from various cultures were identified through literature searches and previous professional interactions. The selection criteria included diversity in cultural backgrounds and established clinical experience. Each HCP was approached to determine their utilization of the CDED in clinical practice. Additionally, they were asked to report on clinical cases demonstrating their experience with CDED, providing both a clinical overview and practical insights into how they adapted the diet to accommodate the patient’s quality of life, while maintaining the principles of CDED. Drawing from Damas et al.’s publication, outlining barriers to dietary intervention adaptation among the Hispanic population [21], we tailored our inquiry to address challenges encountered by HCPs in implementing CDED across populations with distinct dietary habits. Clinical cases and discussions on challenges were compiled collectively. All authors participated in reviewing and approving the final manuscript.

## 3. Results

We present eight unique cases from six countries, covering eight difference cultural/ethnic situations across four continents.

### 3.1. The Use of CDED in Korea

Clinical overview: An 11-year-old girl presented with one month of unexplained fever exceeding 38 °C, along with abdominal pain, diarrhea, and weight loss over three months. Initial lab results showed elevated ESR (Erythrocyte Sedimentation Rate) (46 mm/h), CRP (C-reactive protein) (2.63 mg/dL), and fecal calprotectin (FC) >6000 µg/g, indicating severe CD. Endoscopic examination revealed aphthous ulcers in the stomach and duodenum, and colonoscopy revealed a cobblestone appearance with longitudinal ulcers in the right colon and narrowing at the hepatic flexure. The terminal ileum was not observed due to ileocecal valve stenosis. MRE confirmed multi-segmental bowel wall thickening in the jejunum, ileum, and ascending colon, without evidence of strictures or fistulas. She opted for dietary therapy with CDED and PEN (partial enteral nutrition). After 8 weeks, her symptoms improved, her PCDAI (pediatric Crohn’s Disease activity index) reduced from 47.5 to 12.5, her CRP improved from 2.6 g/dL to 0.3 mg/dL and her FC reduced from <6000 µg/g to 1261 µg/g. Transitioning to phase 2 for 4 weeks led to an additional improvement in inflammatory markers (CRP 0.06 mg/dL and FC 630 µg/g). At 24 weeks, her labs further improved to CRP 0.5 mg/L, and her FC was 19 µg/g. She showed minimal inflammation on repeat colonoscopy, with scar formations and luminal narrowing in the ascending colon.

#### Adapting the Diet to Korean Cuisine: Addressing Challenges and Implementation Strategies

To adapt the CDED in Korea, we worked closely with dietitians to create a Korean version of the CDED protocol (Table 3). Our primary focus was on the adherence to the fundamental principles of CDED, while customizing it to align with the Korean diet during consultations. As with other cuisines, Korean cuisine incorporates a variety of sauces, and utilizes diverse cooking methods. Given that rice is a staple food in Korean cuisine, unlike cultures where flour is a dietary staple, limiting flour posed no significant challenge. However, there were some challenging parts, as many Korean dishes rely on seasonings like soy sauce and sesame oil, while using fewer herbs such as oregano. This required patients to season most of their foods with salt and pepper during the diet, which was a big challenge in maintaining the diet.

Moreover, Korean cuisine tends to consist of combinations of rice, soup, and two to three side dishes rather than a main dish, making it challenging to prepare every meal in a CDED format. Additionally, Korean adolescents tend to enjoy spicy foods, which often contain a lot of seasoning, red pepper flakes, and red pepper paste. To address this issue, a dedicated dietitian provided meal plans and ideas for soups and side dishes that are not necessarily spicy foods while encouraging a diverse menu to assist patients in maintaining their diet.

Another challenge in implementing the CDED was not directly related to the dietary culture or limited to CD, but rather concerned the fact that many Korean schools prepare school lunches for the children. Consequently, having to prepare a separate packed lunch could be burdensome for parents, and some patients experience relative psychological discomfort due to the inability to share the same meals as their friends, as we see with other diseases requiring dietary restrictions, such as celiac disease or food allergies; however, most of the limitations in our case are temporary, which we emphasized during discussions with the family. In our personal experience, we observed that compliance with EEN or CDED during the COVID-19 pandemic was significantly higher compared to both pre-pandemic and post-pandemic periods. This observation may highlight the challenges posed by environmental temptations, including in school.

Frequent visits to the outpatient clinic when on the dietary intervention kept them motivated and helped them to maintain the diet.

As a result of these adaptations, we have seen the CDED being successfully used in multiple cases, including by some patients who were initially expected to require biologics due to deep ulcers and severe disease activity. These patients wanted to defer the use of biologics due to concerns regarding potential side effects while implementing the CDED in Korea, and this did not pose significant challenges.

### 3.2. The Use of CDED in an Arab Patient from Israel

#### 3.2.1. Clinical Overview

A 23-year-old woman from an Arab background presented with diarrhea, 10 kg weight loss and perianal fistula. She was treated with anti-TNF for 7 months. A secondary loss of response occurred, with initial lab results showing elevated CRP (11.85 mg/dL), FC of 4370 µg/g, and decreased albumin levels of 3.05 g/dL, indicating moderate to severe CD (Montreal classification: L3B1p). Endoscopic examination revealed terminal ileum disease with noticeable inflammation and a cobblestone appearance, as well as longitudinal large and linear deep ulcers. The patient opted for dietary therapy with CDED without PEN. By week 6, the symptoms improved; the frequency of bowel movements decreased, and abdominal pain diminished. By week 12, the symptoms continued to improve, and diarrhea ceased. After 24 weeks, CRP improved to 1.16 mg/dL, FC reduced to 914 µg/g, and albumin improved to 3.84 g/dL.

#### 3.2.2. Adapting the Diet to Arabic Cuisine: Addressing Challenges and Implementation Strategies

Residing in Israel and of a Muslim Arab descent, the patient faced several communication and cultural challenges during her participation in the CDED diet. Language emerged as a significant hurdle, as the patient and her mother were not fluent in Hebrew. Given her father’s role as a translator, communication with healthcare providers, who primarily spoke Hebrew, relied on his translation into Arabic. The instructions on the diet were given in two languages, Hebrew and Arabic. Despite this barrier, the patient felt comfortable seeking clarification and participating actively in her treatment. Although conversations with the dietitian occurred via translation, the patient expressed a preference for direct communication in Arabic. However, this limitation did not deter her engagement or motivation, as she understood the rationale behind the diet and observed improvements in her symptoms. The socioeconomic barriers, such as healthcare costs and food accessibility, were minimal due to Israel’s public healthcare system and the affordability of CDED-compliant foods. However, cultural cuisine aspects posed considerable challenges, particularly regarding traditional family meals and religious observances. In Arab culture, family meals are central, often featuring traditional dishes like beef, lamb chops, and pita bread, and they are central for gatherings (Table 4). During the diet’s initial phases, the patient’s mother prepared separate meals for her while the family ate traditionally. During phase 2, Ramadan began, affecting the patient’s adherence, as fasting during the day and traditional meals at night altered her dietary routine. In hindsight, the patient said she would have preferred starting the diet after Ramadan to align with cultural practices more effectively. Presently, 6 months after the initial dietary change, she still adheres to a Mediterranean diet, avoiding processed foods and sugary drinks. To enhance patient guidance and monitoring, offering culturally tailored recipes in Arabic may foster greater adherence and satisfaction among Arab patients undergoing dietary therapy. Introducing the Modulife platform in Arabic has been beneficial, offering recipes and dietary guidance in patients’ native language. Currently, the app provides comprehensive information on CDED through a website and app, offering handouts, diverse global recipes, and optional meal plans in 10 languages, including Arabic.

### 3.3. The Use of CDED by a Hispanic South American Patient from Spain

#### 3.3.1. Clinical Overview

A 12-year-old girl, post-appendectomy, presented with deep ulcers, with a cobblestone appearance of the ileum shown by the endoscopy and elevated CRP (1.4 mg/dL) and FC (309 µg/g). Despite no weight loss or pain, stool consistency changed. Physical examination showed pale skin-mucosa. Additional tests revealed low albumin (3.4 g/dL), and elevated CRP (1.5 mg/dL), ESR (22 mm/h), and FC (543.1 µg/g). Imaging by MRE classified CD (Paris classification: A1L1B1G0) with a wPCDAI (weighted Pediatric Crohn’s Disease activity index) of 25, suggesting mild disease. After 6 weeks on the CDED, wPCDAI improved to 12.5, accompanied by good adherence to the first phase, and the patient continued to phase 2. Despite good motivation, after 12 weeks, clinical remission was not achieved (CRP: 2.4 mg/dL, ESR: 22 mm/h, FC > 2200 µg/g). Biological treatment was planned.

#### 3.3.2. Adapting the Diet to Colombian Cuisine: Addressing Challenges and Implementation Strategies

The patient’s Colombian heritage and ingrained culinary customs initially posed challenges to adhering to the prescribed CDED. Staples like “arepas” and red meat are integral to families’ traditional diets, raising doubts about their ability to comply with dietary restrictions. To address these concerns, the treating dietitian provided tailored guidance and support. They adjusted traditional dishes, offered assistance in meal planning, and introduced modified recipes using alternative ingredients like rice flour for arepas (Table 5). These adaptations helped bridge the gap between cultural dietary preferences and therapeutic requirements, fostering confidence in the patient and her family. Regular follow-up consultations allowed the dietitian to monitor adherence and address emerging challenges. Through ongoing communication, modifications to recipes and meal plans were made as needed, ensuring continued compliance and patient satisfaction. Despite initial apprehensions, the patient successfully integrated the CDED into her daily routine. With minor adjustments and ongoing support, adherence to the therapeutic diet became manageable. Importantly, the dietary modifications not only supported disease management, but also led to improvements in overall dietary habits and lifestyle choices.

This case underscores the significance of culturally sensitive dietary interventions in the management of CD. By acknowledging and addressing cultural dietary preferences, healthcare providers can facilitate patient adherence to therapeutic diets like CDED, ultimately improving treatment outcomes and enhancing quality of life. Through personalized support and ongoing collaboration, patients can navigate cultural dietary challenges effectively, paving the way for successful disease management and holistic well-being.

### 3.4. The Use of CDED in a Patient of Turkish Descent from The Netherlands

#### 3.4.1. Clinical Overview

A 13-year-old boy of Turkish descent presented with persistent slimy and mushy diarrhea, along with elevated FC (775 μg/g), with colonoscopy showing macroscopic inflammation of the stomach, ileum and caecum, and MRI depicting wall thickening of the terminal ileum, matching with ileitis appropriate for the diagnosis of CD. Despite initial plans to start the CDED + PEN during the summer holidays in Turkey, during which time he and the parents assumed it would be easier, the therapy was not initiated. Instead, the patient opted for a “light version” of the diet, which proved ineffective in managing his symptoms. With guidance from the dietitian and gastroenterologist, he resumed full CDED + PEN nutritional therapy, experiencing initial relief but later relapsing. Two weeks into phase 1, his symptoms recurred, but with guidance, he continued the therapy. Four weeks into the diet, his FC decreased to 197 µg/g. After 3 months on phase 3 with PEN, his FC rose to 578 µg/g, prompting a return to phase 2. A month later, his FC reached 779 µg/g and budesonide was initiated. At 14.5 years, lacking motivation, he ceased the diet but continued with the one portion formula, despite his mother’s encouragement. During the holidays in Turkey, where he consumed a diet rich in fresh, additive-free foods, he adhered to Islamic dietary principles and experienced positive outcomes, and the patient expressed a desire to continue this dietary pattern in The Netherlands as well. With continued monitoring and adjustments, including being able to reduce and then stop budesonide, the patient achieved remission. This highlights the significance of culturally sensitive dietary interventions and patient preferences in optimizing treatment outcomes for CD.

#### 3.4.2. Adapting the Diet to Turkish Cuisine: Addressing Challenges and Implementation Strategies

The patient’s Turkish heritage and Islamic dietary preferences posed initial challenges to adhering to the prescribed CDED. To bridge the gap between cultural dietary preferences and therapeutic requirements, the treating dietitian provided tailored guidance and support. They made adjustments to traditional dishes, offered assistance in meal planning, and introduced modified recipes using alternative ingredients. Both the dietitian and physician engaged in an open discussion regarding the initiation of the diet, and the feasibility of waiting and adjusting the diet during vacation. Vacations often present unique challenges, as food availability may be limited, and patients may struggle to control their dietary options.

However, there could also be positive opportunities, such as in this case, when experiencing the ease of following the Mediterranean diet during a family holiday in Turkey rather than at home in The Netherlands, which significantly impacted attitude and motivation. Instead of waiting until after a vacation, dietitians should collaborate with patients to proactively plan how to manage their diet in the destination country and adapt to different foods.

In some cases, if the patient’s condition permits, it may be advisable to recommend starting phase 1 of the diet for 1–2 weeks before the vacation. During the vacation, patients can enjoy local foods (attempting to follow some of the CDED principles), and upon returning home, they can resume phase 1 for an additional 1–2 weeks before transitioning back to their regular diet, whether it be phase 3 or the Mediterranean diet. Dietitians should familiarize themselves with the patient’s food culture, seeking common ground with the prescribed diet to promptly identify challenges and provide alternatives. Family members can assist in refining recipes and adapting the diet to align with family traditions.

Regular follow-up consultations allowed the dietitian to monitor adherence and address emerging challenges, ensuring continued compliance and patient satisfaction. Despite initial apprehensions, the patient successfully integrated the CDED into his daily routine with minor adjustments and ongoing support. Importantly, the dietary modifications not only supported disease management, but also led to improvements in overall dietary habits and lifestyle choices. This case underscores the significance of culturally sensitive dietary interventions in the management of CDED, facilitating patient adherence to therapeutic diets like CDED, and ultimately improving treatment outcomes and enhancing quality of life. Through personalized support and ongoing collaboration, patients can effectively navigate cultural dietary challenges, paving the way for successful disease management and holistic well-being.

### 3.5. The CDED in a Religious Jewish Patient from Israel

#### 3.5.1. Clinical Overview

An 11.8-year-old male, diagnosed with CD in the terminal ileum a year earlier, presented with symptoms of abdominal pain, diarrhea, and blood in the stools. Despite being on immunomodulators treatment, his dietary habits, typical of his ultra-orthodox religious Jewish community, included a high consumption of ultra-processed foods, common in certain Jewish populations. Upon recommendation of anti-TNF, the family opted for dietary therapy, initiating a six-week trial with the CDED. The patient demonstrated significant symptom improvement and favorable improvement in inflammatory markers and nutritional parameters after completing the initial phase of the diet. He did not have abdominal pain, diarrhea, or blood in his stools. Additionally, there was an improvement in inflammatory markers and nutritional parameters. After a year and a half, the patient no longer required anti-TNF therapy. However, Azathioprine was switched to oral Methotrexate once a week due to non-adherence.

#### 3.5.2. Adapting the Diet to Religious Jewish Cuisine: Addressing Challenges and Implementation Strategies

Despite the initial success of the dietary intervention, the patient encountered difficulties in adhering to the prescribed regimen, particularly during the Passover holiday. As part of his religious observance, he was not allowed to consume legumes and rice during the week of Passover, posing challenges in adhering to the dietary restrictions. Additionally, he expressed a desire to partake in traditional Passover customs, including the consumption of matzah (unleavened flatbread) and a small amount of grape juice. Symptomatically, he felt well; however, his CRP and ESR levels increased again. It was decided to start two weeks of EEN followed with phase 1 of the diet with 50% PEN, and to accommodate his religious observance and traditional Passover customs. He then continued into phase 2 with adaptations, adding more potatoes and nuts. Collaboration with the patient and his family resulted in the development of suitable recipes. The adjustments allowed the patient to participate in religious observances while managing his condition effectively.

Meeting the kashrut requirements according to Jewish tradition presents a challenge, particularly during the initial phase of the diet involving a combination of 50% PEN with a dairy-based formula. To tackle this hurdle, the dietitian engaged in discussions with the family. Together, they brainstormed solutions such as adjusting and altering meals and scheduling formula intake during school hours.

Another challenge was the necessity of careful planning and preparation for dietary modifications, which can be burdensome, especially for a large family characteristic of this community. To address this challenge, the responsibility for meal planning and preparation was shifted to the patient, with the support of his parents. The patient was encouraged to actively participate in the preparations, while the parents were there to offer support and provide positive reinforcement. Finally, the family faced financial challenges due to the diet therapy. To address this, the parents were guided by the dietitian to purchase affordable ingredients and seasonal fruits and vegetables. Additionally, they were referred to a hospital social worker for further assistance.

The transition to the phase 3 maintenance diet was made after his CRP decreased to 0.8 mg/dL. With dietitian guidance, monitoring, and support, he succeeded in adhering to the diet. This emphasizes the potential impact of flexible dietary modifications even in populations characterized by a high consumption of ultra-processed foods and specific religious dietary restrictions.

### 3.6. The Use of CDED among Patients from Argentina

#### 3.6.1. Clinical Overview Case 1

A 14-year-old female of German descent, residing in a rural Argentinian town with a population of 350 inhabitants, had a history of celiac disease at age 2. At age 14 she was referred to a tertiary public hospital in Buenos Aires for suspected IBD. She presented with fever, polyserositis, hematochezia, and vomiting, and her blood tests indicated anemia (hemoglobin 5.6 g/dL), hypoalbuminemia (2.3 g/dL), coagulopathy, and elevated CRP (4.59 mg/dL). The endoscopic findings revealed erosions in the duodenum, and multiple ulcers with fibrin deposits in the colon and terminal ileum, confirming chronic inflammation. Nutritional assessment showed acute undernutrition (BMI 17.6 kg/m^2^, z-score: −1.03). Treatment commenced with high-dose corticosteroids and TPN due to oral intolerance, then progressed to EEN; due to a lack of acceptance of EEN, the patient started CDED + PEN as a bridging treatment to anti-TNF. She developed a posterior reversible encephalopathy syndrome as a complication of corticosteroid therapy, requiring antihypertensive medication and rapid corticosteroid tapering. The dietary treatment was well tolerated, leading to symptom resolution within 6 weeks (PCDAI: 0). Subsequent blood tests indicated significant improvement in inflammatory markers: CRP 1.7 mg/dL, hemoglobin 14.6 g/dL and albumin 4.6 g/dL. The patient progressed through the dietary treatment phases, maintaining clinical remission at weeks 12, with Hb 14.4 g/dL, CRP < 0.6 mg/dL, and albumin 5 g/dL. Menstruation returned, fecal calprotectin decreased to 268 µg/g, and capsule endoscopy at 24 weeks showed no mucosal inflammation in the duodenum and ileum.

#### 3.6.2. Clinical Overview Case 2

An 11-year-old boy presented with a three-month history of diarrhea, decreased appetite, and weight loss. Laboratory tests revealed anemia (Hb: 10.5 g/dL), hypoalbuminemia (3.2 g/dL), and an ESR of 27 mm/h, indicating moderate disease activity (PCDAI: 30). His FC was notably elevated, at 720 µg/g. Endoscopic evaluation confirmed CD, with characteristic findings of chronic inflammation. The patient exhibited acute malnutrition, being in the 56th percentile for height (148 cm) and the 20th percentile for weight (33 kg) for his age and sex, and his BMI was 15 kg/m^2^ (z-score −1.39). Despite previous corticosteroid recommendations, the family sought alternative treatments.

Following the diagnosis of moderate CD, the patient underwent a six-week treatment of EEN, followed by phase 1 of the CDED combined with PEN. This took place during the COVID-19 pandemic confinement period, and the family made a chart and daily videos to enable them to carry out the treatment successfully. Most consultations were conducted virtually so as to enhance compliance. At week 6 the patient achieved clinical remission, with a weight gain of 3 kg and a reduction in FCP to 420 µg/g. At 12 weeks, he remained in clinical remission, with further weight gain (5.5 kg) and normalized inflammatory parameters. However, during the transition to phase 3 of the diet, the patient experienced symptomatic relapse after indulging in ultra-processed foods, highlighting the importance of dietary adherence. At one-year after treatment initiation, the patient maintained clinical remission but reported dietary lapses, resulting in elevated inflammatory markers. Reverting to a gluten-restricted diet led to a decline in FCP to 59 µg/g. Despite a brief relapse eighteen months later, the patient responded well to the re-initiation of CDED + PEN, followed by biological therapy. Currently, three years after onset, he remains in clinical remission on anti-TNF therapy, with sustained improvement in nutritional status and laboratory parameters (PCDAI 0, weight 62 kg (70 centile), height 170 cm (75 centile), laboratory normal, FC 48 µg/g).

#### 3.6.3. Adapting the Diet to Argentina: Addressing Challenges and Implementation Strategies

In Latin America, particularly Argentina, PIBD is on the rise [22], with an increasing trend in CD [23]. Safety concerns and growth impairment are major challenges in CD treatment. Addressing these concerns, the CDED was introduced in Argentina. Even if most of the suggested CDED foods are available in Argentina, certain cultural and dietary habits pose obstacles, with many families accustomed to a Western diet high in ultra-processed foods. Many families reported a typical Western diet prior to treatment, characterized by the frequent consumption of fried food, ice cream, and “junk food”. This presents an initial obstacle when introducing the dietary regimen, mirroring challenges observed in other regions. In Argentina, red meats are more commonly consumed than white meats like chicken and fish, with fish intake being occasional or non-existent. Dairy consumption is prevalent, posing challenges for dietary restrictions as natural yogurts are reintroduced in the later phases of CDED. Gluten-free products are available but costly, limiting accessibility. Encouraging fruit and vegetable consumption meets resistance due to fiber concerns and entrenched beliefs. Patients often desire wheat-based foods in phase 2, but alternatives like corn or legume flour are viable options. Additionally, olive oil is limited and expensive, with sunflower or corn oil being more common choices. Therefore, further efforts are needed to ensure the successful implementation of CDED in Argentina.

In Argentina, food carries a profound cultural significance, permeating social and familial gatherings alike. For the pediatric patients, navigating school lunches and participating in communal events presented significant hurdles. To address these challenges, we have engaged in ongoing communication with schools, providing recommendations to ensure dietary compatibility. Additionally, we offer simple, adaptable recipes for social occasions, empowering patients to maintain their dietary regimen while partaking in festivities (Table 6). Over the past five years, more than 30 pediatric patients with CDED were treated at a single center in Argentina, with careful oversight from a multidisciplinary team comprising gastroenterologists and dietitians.

Prior to commencing treatment, patients and their families undergo comprehensive consultation, during which they receive detailed dietary guidance tailored to their needs. This includes informational materials such as infographics, meal charts, and recipes, all presented in Spanish for accessibility. Moreover, patients are equipped with a dedicated smartphone application to facilitate continuous communication with the medical team, ensuring prompt support and guidance throughout their treatment journey. Initial interviews with patients are pivotal, serving as a platform to address concerns and instill confidence in the efficacy of the prescribed diet. Economic constraints pertaining to the affordability of the formula are tackled through various strategies, including group support initiatives and recommendations for alternative formulations. Despite these inherent challenges, the majority of families successfully integrate the dietary regimen into their lifestyles, resulting in favorable outcomes and improved quality of life for the patients.

### 3.7. The CDED in a Patient of Indian Descent from Canada

#### 3.7.1. Clinical Overview

A 7-year-old girl living in Edmonton, Canada was diagnosed with CD after experiencing several weeks of abdominal pain and bloody diarrhea up to 15+ times daily with nocturnal stooling. She lost 7% of her bodyweight during the month before her IBD diagnosis. Diagnostic endoscopy revealed a small perianal fistula and severe left-sided colitis with deep ulcers. Following diagnosis, she commenced EEN as her initial management while being worked up for biologic therapy. At the follow-up visit two weeks after, the family showed frustration/concern with continuing EEN treatment as the patient really missed food. At that point, the team advised the family to introduce the CDED diet together with PEN. In addition to dietary modifications, she received anti-TNF for her perianal and luminal disease shortly after diagnosis, with immunomodulators later added to her treatment regimen. At week 0 of CDED, her PCDAI score was 52.5, her CRP level was 8.5 mg/dL, and her FC count was 4622 µg/g. Over 24 weeks, a significant improvement was noted, with her PCDAI decreasing to 20 at week 6, further dropping to 15 by week 12, and remaining at 15 by week 24. Similarly, her CRP levels decreased substantially from 8.5 mg/dL at week 0 to 1.22 mg/dL at week 6, then to 0.25 mg/dL at week 12, and 0.22 mg/dL at week 24. Her FC counts also showed a marked decline, reducing from 4622 µg/g at week 0 to 998 µg/g at week 6, and then to 517 µg/g at week 12, and 419 µg/g at week 24, indicating her positive response to this combined treatment.

#### 3.7.2. Adapting the Diet to Indian Cuisine: Addressing Challenges and Implementation Strategies

The family originally came from India and was still eating predominantly traditional food at home. When the CDED was first discussed, it posed a significant challenge for her family, who adhered to a traditional vegetarian diet due to cultural and religious reasons. Additionally, her egg allergy further complicated dietary adjustments based on the CDED. To ensure adequate protein intake, the family agreed to incorporate chicken into her diet while on the CDED, although it was not a favored option. The family was provided with access to the Modulife app for recipes using chicken and other ingredients that the family was less familiar with. However, due to ongoing symptoms, chicken was later removed from her diet as the family was worried that it might act as a trigger for her symptoms. To address this, we worked with the family to integrate other traditional Indian foods such as dal, chapati, and homemade yogurt into her diet, ensuring compatibility with the CDED guidelines. Despite these efforts, the patient’s limited acceptance of CDED-friendly foods, necessitated modifications to her liquid meal replacements, to provide approximately 50% of her calorie needs throughout the CDED phases. Towards the end of the CDED phase, the family was hesitant to expand her food options due to fear of symptom recurrence, which presented another challenge. Reassurance and encouragement were crucial in overcoming this reluctance and allowing her to gradually reintroduce foods beyond the CDED diet.

## 4. Discussion

**Figure 1:**
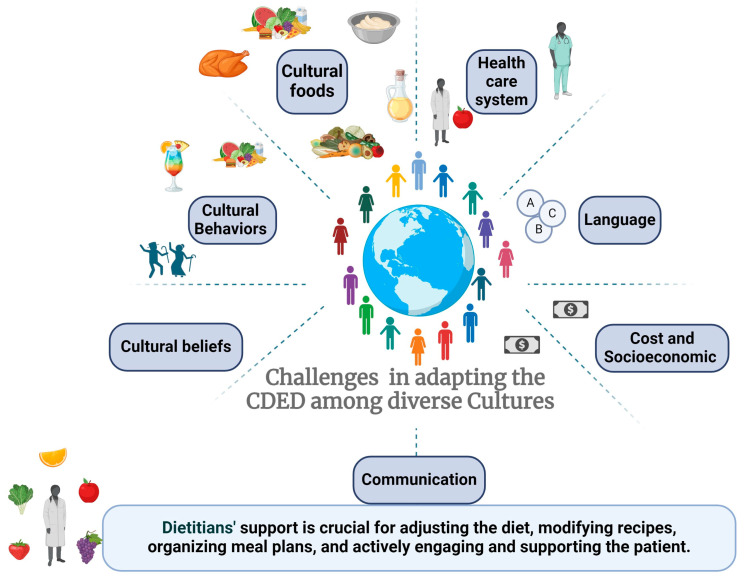
Multifaceted challenges in adapting the Crohn’s Disease Exclusion Diet across diverse cultural contexts.

In our case study, we shed light on the complexity yet feasibility of adapting and implementing the CDED across different cultures. Acknowledging that various communities may require unique considerations, we address the potential challenges diligently. We outline these challenges in Figure 1 and offer optional solutions in Table 7.

We documented our experience with the CDED across a spectrum of CD phenotypes, ranging from mild to severe, either in combination with medications or as a monotherapy strategy, as commonly observed in our clinic. We detailed the clinical management and progression, along with the challenges and solutions provided by healthcare providers, particularly dietitians. We underscored the crucial role of dietitians in disease management, especially when diet plays a pivotal role in treatment. Diet is multifaceted and intertwined with mood, social events, lifestyle, and more. Our aim as healthcare providers is to enable patients to maintain their lifestyle as much as possible while guiding them on how to adjust their diet effectively to alleviate the burden of change. This involves providing recipes, educating patients, and negotiating when necessary to ensure their well-being. Table 3, Table 4, Table 5 and Table 6 illustrate examples provided by dietitians of how to modify traditional meals to align with the CDED. The challenges encountered underscore the need for collaborative approaches.

Recent years have presented dietary shifts, as many patients have moved away from culturally specific cuisines and embraced the industrialized Western diet [24]. This preference can be attributed to the economic advantage and easy accessibility of Western diets, as well as globalization and marketing. This shift and its effect on health underscore the importance of promoting healthier dietary habits, regardless of IBD. CDED adherence, in turn, enhances patients’ dietary behaviors and habits, as was shown by a group from Spain [13]. Over 52 weeks of following the CDED, a significant improvement in dietary habits was seen.

It was recently shown that a high-quality diet early in life may lower the risk of subsequent IBD [25]. In addition, it was reported that upon immigration, many Hispanic patients shift to less healthy diets, embracing local cuisine [26]. Traditional foods often provide healthier options compared to processed Western diets, and therefore one of our aims is to uphold traditional culinary practices while guiding patients in adjusting local cuisine to better accommodate their health needs during the disease [27]. There is a growing body of evidence supporting the efficacy of the CDED in inducing remission among patients with CD, particularly those with mild to moderate disease. Expert reviews suggested the possibility of extending its application to a broader spectrum of CD cases [28]. With the recent endorsement of CDED as an alternative therapy to EEN in the ESPEN guidelines [2], its acceptance and utilization have gained traction, offering a legitimate therapeutic option for clinicians and patients alike across the world. In addition, it is recognized in several reviews by other leading groups as an appropriate treatment for mild-to-moderate disease [29,30,31]. This recognition has catalyzed interest globally, as evidenced by initiatives such as the development of an optimal care pathway set out by dietitians from the Crohn’s and Colitis Australian Network, specifically tailored for the clinical implementation of CDED among adult populations in Australia [32]. These developments underscore the evolving landscape of dietary interventions in CD management, and highlight the expanding role of CDED as a viable treatment strategy with potential applicability across various clinical scenarios.

As the CDED follows a standardized approach, its ingredients are selected to be affordable and accessible worldwide. However, traditional or local foods were not initially considered. Initially, we gathered recipes from local patients, compiling them into a recipe booklet shared with all participants. This approach was replicated during the first RCT with Canadian patients, where a local recipe booklet and support system were developed. Subsequently, the Modulife program was created, offering a website and app with valuable information on CDED, including handouts, global recipes, and meal plans in 10 languages. Engaging nearly 7000 patients across 60 countries, Modulife also provides educational programs for healthcare providers, facilitating comprehensive training in CDED implementation. While healthcare providers from various countries actively utilize this platform, the pivotal role of educated dietitians in disseminating these resources and guiding populations through dietary transitions deserves greater promotion.

Understanding the principles of CDED is essential for a successful experience. A group from The Netherlands attempted to implement CDED without adhering to guidance or educational materials, resulting in poor compliance and weight loss [33]. Knowledge, adherence, and support provision are key aspects for successfully implementing dietary therapy. The initial studies investigating the role of CDED employed a standardized diet without deviating from the dietary instructions [4,5,8]. This approach aimed to assess whether the strategy could effectively induce remission. After demonstrating efficacy, adjustments to the diet became feasible, while ensuring adherence to the core principles and the continued exclusion of key components. Similar methodologies were applied to the low-FODMAP diet, which initially utilized strict principles [34] later replicated by other groups to alleviate symptoms in IBS [35], indicating the diet’s effectiveness.

In recent years, we have observed gentler versions and personalized adaptations of the diet, reflecting progress over time [36]. However, it is crucial to maintain the core principles to preserve the essence of CDED. Excessive adjustments may result in a diet diverging from the original program, potentially leading to varied outcomes and misconceptions about CDED’s efficacy. A recent study from Slovenia described a modified CDED with ingredients typical to their region, deviating significantly from the standard protocol [37]. While such modifications may suit local preferences, they could dilute the effectiveness of the diet and undermine its true potential. Thus, caution is warranted in labeling such variations as CDED, as they may not align with its intended design and could impact research outcomes. Damas et al. emphasized cost as a barrier to dietary adoption [21], yet the CDED comprises affordable components. A study from Spain revealed that CDED expenses aligned with average daily food costs for Spanish individuals [38]. Another study from Israel suggested that CDED could serve as a monotherapy for maintaining remission up to week 24 without supplementary medication, potentially reducing medication dependence in some patients and enhancing cost-effectiveness [8]. However, further comprehensive data are needed to confirm its long-term maintenance role. Conversely, a study from the United Arab Emirates found that cost was not a significant barrier for most patients adopting CDED [39]. They noted that while the CDED was more tolerable than EEN, maintaining compliance with this strict diet was challenging, especially for many children who were picky eaters. Nevertheless, over 50% of participants persisted with the diet beyond week 12.

This study’s findings are limited by selection biases of healthcare providers based on personal connections, potentially skewing the representation of challenges faced. The cases presented may not encompass all cultural contexts, and additional, unexplored challenges in healthcare delivery could exist beyond those discussed. Additionally, due to the study design, statistical analyses were not feasible; only case descriptions based on personal experiences are presented. The study also lacked a systematic review of current evidence, focusing instead on personal experiences. Our study prioritized the experiential and practical aspects of healthcare challenges. We explicitly emphasize that our findings are not intended to establish broad scientific conclusions, but rather to share nuanced observations and experiences. By focusing on these insights, we aim to contribute to the ongoing discourse, and potentially guide future research and clinical practices.

## 5. Conclusions

In conclusion, our study emphasizes the crucial role of healthcare providers, particularly dietitians, in tailoring diets to suit diverse cultural backgrounds. We recognize that, regardless of ethnicity, every CD patient must adjust their dietary pattern when starting CDED, and cultural foods and habits can sometimes make this process more challenging. We provide solutions for successful adherence to the CDED across different ethnicities and countries, highlighting the pivotal role of dietitians in guiding patients through dietary adjustments. This underscores the potential of dietary interventions to transcend cultural barriers and benefit diverse communities. The success of the CDED in accommodating various cultures serves as a promising model for future research. The continued exploration of these strategies, with active involvement from dietitians, holds immense promise for enhancing the health and well-being of underrepresented communities affected by IBD.

## Figures and Tables

**Table 1:** The research experience of CDED in different countries.

Country	Study Reference	Study Population	Diet Adaptation/Cultural Issues
Israel	Sigall-Boneh 2014 [4], Levine 2019 [5], Stein 2022 [7], Yanai 2022 [8], Isakov-Fliss 2003 [9]	Children and adults with active CD	The studies conducted in several centers across Israel reflect diversity and various ethnicities within the population. Additionally, the population exhibits diversity in religious beliefs.
USA and Canada	Levine 2019 [5], Stein 2022 [7]	Children and young adults with CD in active disease and in remission	Cultural characteristics include high consumption of fast foods and ultra-processed foods, necessitating the search for suitable alternatives and the adoption of home cooking and meal preparation.
Poland	Matuszczyk 2022 [10], Szczubelek 2021 [11]	Children and adults with active CD	Modulife program with recipes and dietetic consultation to assess tolerance and adherence to the diet was sufficient when following the regular CDED guidelines and avoiding ultra-processed foods. Only the amount of obligatory products was increased if necessary. No other adaptations were needed.
Spain	Jijon Andrade2023 [12], Martin-Masot 2023 [13]	Children with mild–moderate CD	Following the diet resulted in a reduction in ultra-processed food intake, high diet quality, and high adherence to the Mediterranean diet. No specific cultural or ethnical aspects, or CDED diet adaptations, are mentioned.
Croatia	Niseteo 2022 [14]	Pediatric CD patients	No adaptations to CDED were described.
Slovenia	Urlep 2020 [15]	Pediatric CD patients	Sometimes AID-CD (anti-inflammatory) is offered, a diet CDED modification with Slovenian products.
Italy	Scarallo 2021 [16], Scarallo 2024 [17]	Mild to moderate luminal–colonic CD patients	The CDED was well tolerated, and adherence to the diet regimen was good. No specific cultural or ethnical aspects, or CDED diet adaptations, are mentioned.
Argentina	Arcucci 2023 [18]	Asymptomatic children with CD and elevated FCP on medical treatment	Compliance was not a problem in the CDED + PEN group, and no diet adaptations are mentioned.
New Zealand	Wall 2024 [19]	Adult CD who required elective gastrointestinal surgery	No adaptations to CDED are described.
Australia	Landorf [20]	Children with active CD	No cultural adaptations are described, but adjustments were made for vegan diets and cow’s milk allergy.

CD—Crohn’s Disease; CDED—Crohn’s Disease Exclusion Diet; PEN—Partial Enteral Nutrition.

**Table 2:** Experiences of each participating center in treating patients with CDED.

Center	Country	Population	Number of Patients Treated with CDED per Year
The E. Wolfson Medical Center	Israel	Children	70–80
Severance Children’s Hospital	Korea	Children	10
Hospital de niños Ricardo Gutierrez	Argentina	Children	3–5
Hospital Italiano de Buenos Aires	Argentina	Children	20–30
Hospital Regional Universitario de Málaga	Spain	Children	60–70
Haemek Medical Center	Israel	Adults	20–30
Emma Children’s Hospital/Amsterdam UMC	The Netherlands	Children Adults	25–35 5–10
University of Alberta	Canada	Children	20–30

**Table 3:** Example of adaptation of a traditional Korean recipe to align with the CDED principles.

Traditional Korean Meal	CDED Adjustment
Original recipe: **Gimbap** Seasoned rice and stuffings such as hams, crabmeat, and bulgogi (beef marinated with soy sauce, sugars, sesame oils, etc) wrapped with seaweed. Usually, sesame oils and sesames are put on top of gimbap.	We replaced seaweed with rice paper. Rice was seasoned with small amount of vinegars and salts. Instead of hams, crabmeats and bulgogi, we recommended chicken and steamed vegetables to be used as stuffings.

**Table 4:** Adaptation of traditional Arabic meals to align with the CDED principles.

Traditional Arabic Meal	CDED Adjustment
**Pita bread**: A staple in Arabic cuisine, often used for dips and sandwiches. Pita is made from yeast and wheat flour.	Substituting yeast and wheat flour with rice flour and olive oil.
**Synia**: Traditional Middle Eastern meat and tahini casserole typically made with lamb or beef.	Using ground chicken breast, potatoes, onions, and tomatoes instead of lamb/beef. For phase 2, incorporating raw tahini and eggplants/cauliflower.
**Baklava**: A dessert of honey-soaked phyllo pastry layered with nuts.	In phase 2, instead of phyllo pastry we used rice paper and Canola oil, and the rest of the ingredients were identical to the original recipe with sugar and honey as the syrup, and walnuts for the filling.

**Table 5:** Example of the adaptation of a traditional Colombian recipe to align with the CDED principles.

Traditional Colombian Recipe	CDED Adjustment
**Arepas** – 2 cups of precooked corn flour – 1 teaspoon of butter – 1 cup grated mozzarella – Typical filling for arepas is shredded beef with cheese	– Substitute precooked corn flour with rice flour – Replace butter with olive oil – Remove mozzarella from the recipe (similar to Venezuelan arepas) During phase 1: – Use shredded chicken breast and avocado or scrambled egg with tomato and onion During phase 2: – Use shredded beef and beans in the filling

**Table 6:** Adaptation of traditional Argentinian meals to align with the CDED principles.

Traditional Argentinian Meal	CDED Adjustment
**Asado**: Traditional meal, very frequent in family gatherings or with friends, as well as at birthdays or festivities, where beef is roasted over firewood	Roasted chicken/fish. Chicken brochettes with vegetables. Roasted vegetables (potato, onion, tomato in phase 1; eggplant, pumpkin, sweet potato in phase 2). From stage 2 onwards, one portion of red beef such as loin or peceto is allowed.
**Pasta**: On Sundays, families often have family lunches that include pasta with wheat flour with stew (tomato sauce with vegetables, red meat and sausages)	Gnocchi recipe (cooked with mashed potato, egg, salt) or rice noodles. For the stew, a chicken stew recipe with chicken and vegetables (tomato, onion, carrot, potato) is usually given
**Traditionnel dessert**: Flan with dulce de leche (caramel sauce)	The family is encouraged to cook the flan at home. It is suggested to use formula to cook the flan and the dulce de leche (12 measures of formula plus 50 g of sugar, 1/2 spoonful of baking soda and 1/4 vanilla stick). The same can be done with other traditional desserts such as rice pudding, pancakes with dulce de leche and custard.
**School lunch**: Usually families send ready-to-eat meals, which can be out of the refrigerator for a few hours, or the children eat the food offered in the cafeteria. The snack time is the most complicated since they usually offer alfajores (wheat flour dough with dulce de leche and chocolate coating), sweet cookies, fried potatoes.	If the family is able to send a meal to school, this option is encouraged, with homemade food preferred. Otherwise, a list of permitted foods will be sent to the school cafeteria for adaptation. Recipes are given for potato and egg salad, vegetable or tuna pie or empanadas (rice flour with water and oil), rice croquettes with vegetables, potato tortilla, chicken nuggets/sausages (chicken, carrot, parsley, egg, rice flour). For snacks we suggest puffed rice or meringues (egg white with sugar, lemon or orange zest), and recipes are offered for apple or lemon cookies, banana muffins, fruit muffins, dried fruits in phase 2, olives/cherry tomatoes/carrot sticks.

**Table 7:** Challenges and proposed solutions in implementing dietary interventions across diverse cultural settings.

Barrier	Relevant Points	Solutions for Healthcare Practitioners
Healthcare system	Lack of multidisciplinary teams and language barriers	Ensure availability of a multidisciplinary team, including a dietitian fluent in the patient’s language. Utilize translation services when necessary to ensure understanding of instructions and rationale. Use international guidelines to advocate for having a dietitian as part of your MDT.
Language	Lack of information in native language	Utilize the Modulife app and supportive materials available in 10 different languages. Build local resources to share materials.
Costs and Socioeconomic	Healthy foods may be more expensive; lack of knowledge regarding healthy diet	Guide patients in selecting affordable, seasonal fruits and vegetables according to recommendations. Educate patients on reading food labels and making informed choices. Advocate for support from the local medical system for potential cost-saving nutritional therapies.
Cultural Behaviors	Food is integral to cultural and social events	Understand cultural foods and gather recipes from patients to share with others. Customize cultural recipes to align with CDED principles, increasing adherence. Recommend more activities not related to foods.
Cultural Foods	Traditional foods may not align with CDED	Prepare special meals, organize events with CDED-friendly foods, and adjust recipes for specific occasions. Emphasize the temporary nature of dietary restrictions.
School meals	School meal programs may not provide CDED-friendly options, lack of control over ingredients and preparation	Collaborate with school administrators and nutrition staff to develop CDED-compliant meal options. Provide guidelines and recipes to schools for preparing suitable meals. Encourage parents to pack CDED-friendly meals for their children. Educate school staff on the importance of dietary adherence for the child’s health.

## Data Availability

Data supporting reported results are available upon request. Data are not public due to ethical reasons.
